# Biomarkers of Collagen Metabolism Are Associated with Left Ventricular Function and Prognosis in Dilated Cardiomyopathy: A Multi-Modal Study

**DOI:** 10.3390/jcm12175695

**Published:** 2023-09-01

**Authors:** Anne G. Raafs, Bouke P. Adriaans, Michiel T. H. M. Henkens, Job A. J. Verdonschot, Myrurgia A. Abdul Hamid, Javier Díez, Christian Knackstedt, Vanessa P. M. van Empel, Hans-Peter Brunner-La Rocca, Arantxa González, Joachim E. Wildberger, Stephane R. B. Heymans, Mark R. Hazebroek

**Affiliations:** 1 Department of Cardiology, Maastricht University Medical Centre, 6229 HX Maastricht, The Netherlands; bouke.adriaans@mumc.nl (B.P.A.); michiel.henkens@mumc.nl (M.T.H.M.H.); job.verdonschot@mumc.nl (J.A.J.V.); c.knackstedt@mumc.nl (C.K.); vanessa.van.empel@mumc.nl (V.P.M.v.E.); hp.brunnerlarocca@mumc.nl (H.-P.B.-L.R.); s.heymans@maastrichtuniversity.nl (S.R.B.H.); mark.hazebroek@mumc.nl (M.R.H.); 2 Cardiovascular Research Institute Maastricht (CARIM), Maastricht University, 6229 HX Maastricht, The Netherlands; j.wildberger@mumc.nl; 3 Department of Radiology and Nuclear Medicine, Maastricht University Medical Centre, 6229 HX Maastricht, The Netherlands; 4 Netherlands Heart Institute (NLHI), 3511 EP Utrecht, The Netherlands; 5 Department of Pathology, Maastricht University Medical Centre, 6229 HX Maastricht, The Netherlands; m.abdulhamid@mumc.nl; 6 Department of Clinical Genetics, Maastricht University Medical Centre, 6229 HX Maastricht, The Netherlands; 7 Program of Cardiovascular Diseases, CIMA Universidad de Navarra and IdiSNA, 31008 Pamplona, Spain; jadimar@unav.es (J.D.); amiqueo@unav.es (A.G.); 8 CIBERCV, Carlos III Institute of Health, 28029 Madrid, Spain; 9 Department of Cardiovascular Research, University of Leuven, 3000 Leuven, Belgium

**Keywords:** dilated cardiomyopathy, fibrosis, cardiovascular magnetic resonance, feature tracking, global longitudinal strain, CITP/MMP-1

## Abstract

Background: Collagen cross-linking is a fundamental process in dilated cardiomyopathy (DCM) and occurs when collagen deposition exceeds degradation, leading to impaired prognosis. This study investigated the associations of collagen-metabolism biomarkers with left ventricular function and prognosis in DCM. Methods: DCM patients who underwent endomyocardial biopsy, blood sampling, and cardiac MRI were included. The primary endpoint included death, heart failure hospitalization, or life-threatening arrhythmias, with a follow-up of 6 years (5–8). Results: In total, 209 DCM patients were included (aged 54 ± 13 years, 65% male). No associations were observed between collagen volume fraction, circulating carboxy-terminal propeptide of procollagen type-I (PICP), or collagen type I carboxy-terminal telopeptide [CITP] and matrix metalloproteinase [MMP]-1 ratio and cardiac function parameters. However, CITP:MMP-1 was significantly correlated with global longitudinal strain (GLS) in the total study sample (R = −0.40, *p* < 0.0001; lower CITP:MMP-1 ratio was associated with impaired GLS), with even stronger correlations in patients with LVEF > 40% (R = −0.70, *p* < 0.0001). Forty-seven (22%) patients reached the primary endpoint. Higher MMP-1 levels were associated with a worse outcome, even after adjustment for clinical and imaging predictors (1.026, 95% CI 1.002–1.051, *p* = 0.037), but CITP and CITP:MMP-1 were not. Combining MMP-1 and PICP improved the goodness-of-fit (LHR36.67, *p* = 0.004). Conclusion: The degree of myocardial cross-linking (CITP:MMP-1) is associated with myocardial longitudinal contraction, and MMP-1 is an independent predictor of outcome in DCM patients.

## 1. Introduction

Myocardial fibrosis is a fundamental process in the pathophysiology of heart failure (HF) and is associated with increased LV stiffness, impaired systolic contraction, and long-term mortality in patients with non-ischemic dilated cardiomyopathy (DCM) [1,2]. Myocardial fibrosis occurs when collagen deposition exceeds degradation. The functional impact of myocardial fibrosis not only depends on the quantity of collagen fiber deposition (mainly type I fibers) but also on the quality of collagen fibers (degree of collagen cross-linking) [3]. We previously showed that the quantity of myocardial fibrosis deposition is reflected by the levels of circulating carboxy-terminal propeptide of procollagen type I (PICP), with increased levels corresponding to poor prognosis in DCM patients [4,5,6,7]. However, the impact of fibrosis quality on cardiac function and prognosis in DCM is unknown. Increased myocardial collagen cross-linking exacerbates resistance to collagen fiber degradation, enhances myocardial stiffness, and impairs signal transmission, leading to impaired myocardial contraction [7]. Recently, a serum biomarker of collagen cross-linking, the ratio of collagen type I carboxy-terminal telopeptide to matrix metalloproteinase-1 (CITP:MMP-1), was found to be inversely related to tissue-assessed myocardial collagen cross-linking in patients with hypertensive heart failure [8]. MMP-1 cleaves CITP (one of the two major cross-link sites of collagen fiber), resulting in collagen fiber degradation [8,9].

Whether these collagen metabolism-related biomarkers are associated with myocardial contraction, function, and prognosis in DCM patients is still unknown. Therefore, the aim of the present study was to investigate the associations between circulating markers of collagen deposition (PICP), collagen cross-linking (CITP:MMP-1), and histological collagen volume fraction (CVF) with cardiac function parameters, and to evaluate whether collagen cross-linking and degradation biomarkers further improve risk stratification in DCM patients.

## 2. Materials and Methods

### 2.1. Study Population

Consecutive DCM patients were prospectively enrolled in the Maastricht Dilated Cardiomyopathy Registry between 2004 and 2017 (n = 928). Patients from this registry who underwent CMR, blood sampling, and EMB within a three-month timespan were included in this study (n = 209, Supplementary Figure S1). The diagnosis of DCM was confirmed using the World Health Organization/International Society and Federation of Cardiology definition, based on a reduced LVEF and an increased LV end-diastolic volume (LVEDVi) indexed to the body surface area (BSA), compared to the published age- and sex-specific reference values [10,11]. In keeping with the guidelines [11,12,13], the exclusion criteria included (i) myocardial infarction and/or significant coronary artery disease (stenosis > 50%, ruled out using coronary artery angiography or computed tomography) and/or presence of infarct patterns of LGE on CMR; (ii) primary valvular disease; (iii) hypertensive or congenital heart disease; (iv) acute myocarditis; (v) arrhythmogenic right ventricular dysplasia; and (vi) hypertrophic, restrictive, or peripartum cardiomyopathy. All patients underwent a clinical diagnostic workup including a medical history interview, physical examination, blood sampling, 12-lead electrocardiogram, CMR, and EMB during or after their first visit to our specialized DCM outpatient clinic. This study was performed according to the Declaration of Helsinki and was approved by the institutional Medical Ethics Committee (Ethic Committee Name: Medisch-ethische toetsingscommissie azM/Maastricht University (METC azM/UM); approval code: 12-4-013). All patients provided written informed consent.

### 2.2. CMR Acquisitions and Analysis

All included patients underwent CMR during diagnostic workup. No patients had prior implantation of an electronic device (i.e., pacemaker, internal cardiac defibrillator, or cardiac resynchronization therapy) at the time of the CMR. CMR was performed using a 1.5T system (Ingenia, Philips Medical Systems, Best, The Netherlands). The acquisition protocol included cine imaging for functional analysis and two-dimensional late gadolinium enhancement (LGE) in the long and short axes of the left ventricle for visual detection of focal fibrosis. Cine images were acquired during end-expiratory breath holds, using a balanced steady-state free precession sequence. LGE imaging was performed 10–15 min after an intravenous bolus of 0.2 mmol/kg body weight of gadolinium-diethylenetriaminepentaacetic acid (Gadobutrol, Bayer, Berlin, Germany). LGE was considered present if it was reproducibly observed in multiple views (i.e., long- and short-axis planes) and extending beyond the localized ventricular insertion areas. Typical RV insertion areas of fibrosis were excluded. LGE quantification was performed using the full-width-at-half-maximum method [14]. 

Feature-tracking strain analyses were performed semi-automatically using a dedicated software (CAAS MR Solutions 5.2.1, Pie Medical Imaging, Maastricht, The Netherlands) by a trained investigator, who was blinded to the outcome (AR). The end-diastolic and end-systolic phases (defined as the largest and smallest LV volume, respectively) were manually selected, after which the software automatically tracked endo- and epicardial contours in consecutive frames of the short-axis view and 2- and 4-chamber long-axis views. Global longitudinal, circumferential, and radial strains were subsequently calculated. To evaluate intra-observer variability, strain analyses were repeated using 25 CMR scans, at least two weeks after the first measurement. The proportional bias of GLS, GCS, and GRS was excluded based on the Bland–Altman analysis, and the intra-observer agreements were optimal (interclass correlation coefficient [ICC] GLS = 0.99, ICC GCS = 0.92, and ICC GRS = 0.99, Supplementary Figure S2).

### 2.3. Biochemical Studies

Blood sampling was performed at the time of the EMB procedure. The samples were aliquoted and kept at −80 °C until measurements were conducted. Serum PICP (Quidel Corporation, San Diego, CA, USA), CITP (USCN Life Science, Wuhan, China) and MMP-1 (GE Healthcare, Tokyo, Japan) were measured using enzyme-linked immunosorbent assay methods. A total of 42 patients showed MMP-1 levels below the analytical detection limit (<1.70 ng/mL). For further analyses, the minimum analytical detection limit of MMP-1 levels was used, which was previously reported as a valid approach for MMP-1 levels with non-detects [15]. The CITP:MMP-1 ratio was calculated by dividing CITP by MMP-1 concentration in molar units as previously described [8].

### 2.4. Endomyocardial Biopsy

All included patients underwent EMB as part of the diagnostic clinical workup. At least six EMB samples were taken from the right ventricular septum via the internal jugular vein using a transcatheter bioptome (Cordis, Miami, FL, USA). In each patient, three specimens were used for immunohistological analysis and three for the detection of viral genomes [16]. Histopathological tests were performed using 4 µm thick tissue sections from the formalin-fixed, paraffin-embedded EMBs, and stained with hematoxylin and eosin, Sirius red, CD3+, CD45+, and C68+. Increased cardiac inflammation was defined as ≥14 CD45, including up to 4 CD68-infiltrating cells/m^2^, in line with the current ESC position statement [17]. CVF was evaluated using five to seven high-power (200×) magnification digital images, covering the total biopsy. In addition, one to two 40× magnification images were acquired per patient for semiautomated analysis (ImageJ version 1.50b, National Institute of Health, Bethesda, MD, USA) [18]. CVF was quantified as percentage of tissue positive for Picrosirius red in the total myocardial area, excluding subendocardial and perivascular areas. The average of the quantification of the different images was considered the final CVF value.

### 2.5. Prognosis

Follow-up data on all-cause mortality, life-threatening ventricular arrhythmias, and HF hospitalization were collected from medical records, municipal population register, and/or telephone contact with general practitioners. The primary endpoint to evaluate the associations between collagen biomarkers and prognosis was defined as a combination of all-cause mortality, HTx, life-threatening arrhythmias, and HF hospitalization. Life-threatening arrhythmias were defined as ventricular fibrillation (with or without implantable cardioverter-defibrillator shock), hemodynamically unstable ventricular tachycardia, or sustained ventricular tachycardia with implantable cardioverter-defibrillator shock.

### 2.6. Statistical Analysis

All variables are displayed as numbers (percentages), mean ± standard deviation, or median [IQR], as appropriate. Normality was evaluated using the Shapiro–Wilk test. The Bland–Altman analysis was performed to evaluate intra-observer variability of the strain measurements and the strength of the variability was assessed using intraclass correlation coefficients based on absolute agreement, using a two-way mixed-effects model. Pearson’s correlation coefficient was used to examine the association between collagen biomarkers, cardiac function parameters, and histological/imaging fibrosis parameters. Covariates known to be predictive of outcome in DCM were included in the adjusted model: age, sex, LVEF, NYHA class ≥ 3, NTproBNP, PICP, and LGE presence [5]. Kaplan–Meier survival curves were estimated and the differences between groups were assessed via the long-rank test. Univariable and multivariable Cox proportional hazards regression analyses were performed to determine the hazard ratio (HR) and subsequent 95% confidence interval (CI). The incremental value of collagen on top of the other predictors was tested using a likelihood ratio test. Statistical analyses were performed using SPSS 26.0 (IBM Corp., Armon, NY, USA) software.

## 3. Results

### 3.1. Patient Characteristics

Clinical characteristics are presented in Table 1. In total, 209 patients were included (Supplementary Figure S1). The mean age at diagnosis was 54 ± 13 years, 65% was male, and approximately two-thirds of patients presented with NYHA class I or II. The mean LVEF was 34 ± 12% and the mean GLS was −10 ± 4%. The median time between CMR and EMB and blood sampling was 30 days [IQR 6–50].

### 3.2. Associations of Collagen Cross-Linking Biomarkers with Myocardial Fibrosis Based on Histology and Imaging

**Figure 1:**
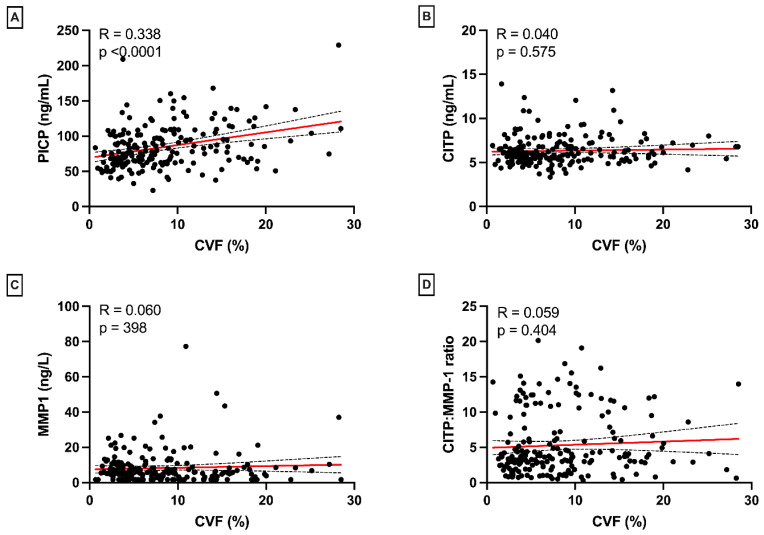
Associations of collagen biomarkers with histological CVF. PICP is correlated with CVF (**A**), but CITP, MMP-1, and CITP:MMP-1 ratio arenot (**B**–**D**). Abbreviations: CVF: collagen volume fraction, PICP: carboxy-terminal propeptide of procollagen type I, CITP: collagen type I carboxy-terminal telopeptide, MMP-1: matrix metalloproteinase-1. Red line = linear regression line. Dotted lines = 95% CI.

**Figure 2:**
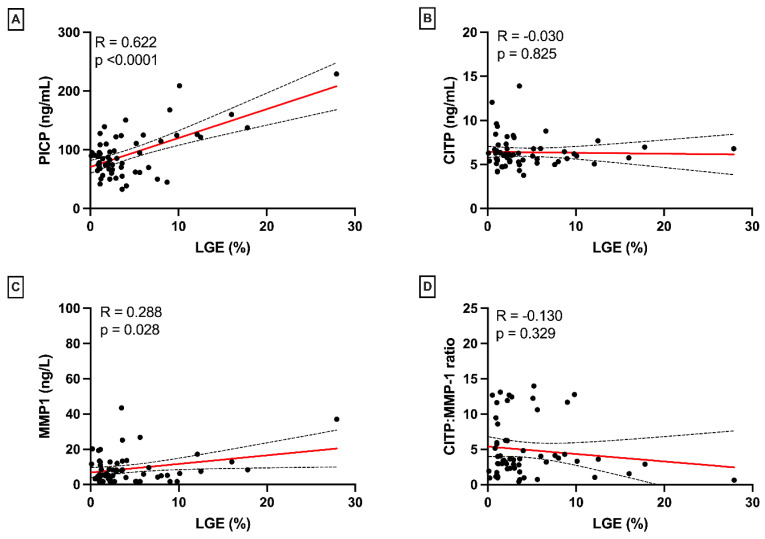
Associations of collagen biomarkers with LGE extent. PICP (**A**) and MMP-1 (**C**) are correlated with LGE extent, but CITP (**B**) and CITP:MMP-1 ratio are not (**D**). Abbreviations: LGE: late gadolinium enhancement, PICP: carboxy-terminal propeptide of procollagen type I, CITP: collagen type 1 carboxy-terminal telopeptide, MMP-1: matrix metalloproteinase-1. Red line = linear regression line. Dotted lines = 95% CI.

We previously showed that PICP levels were significantly correlated with fibrosis based on histology (CVF R2 = 0.17, *p* = 0.001, and LGE extent R2 = 0.39, *p* < 0.001 [5]). However, no correlation was found between the CITP:MMP-1 ratio, reflecting collagen cross-linking (fibrosis quality), and CVF (fibrosis quantity, R = 0.06, *p* = 0.40, Figure 1D), with similar results for CITP (R = 0.04, *p* = 0.58) and MMP-1 (R = 0.06, *p* = 0.40) alone (Figure 1B,C). CITP, MMP-1, and CITP:MMP-1 were not significantly different between patients with and without LGE (*p* = 0.68, *p* = 0.28, and *p* = 0.28, respectively). MMP-1 showed a weak but significant correlation with the LGE extent (R = 0.29, *p* = 0.03, Figure 2C). CITP and CITP:MMP-1 ratio were not correlated with the LGE extent (CITP: R = −0.03, *p* = 0.83, and CITP:MMP-1: R = −0.13, *p* = 33, Figure 2B,D). 

### 3.3. Associations of Collagen Biomarkers with Cardiac Function

**Figure 3:**
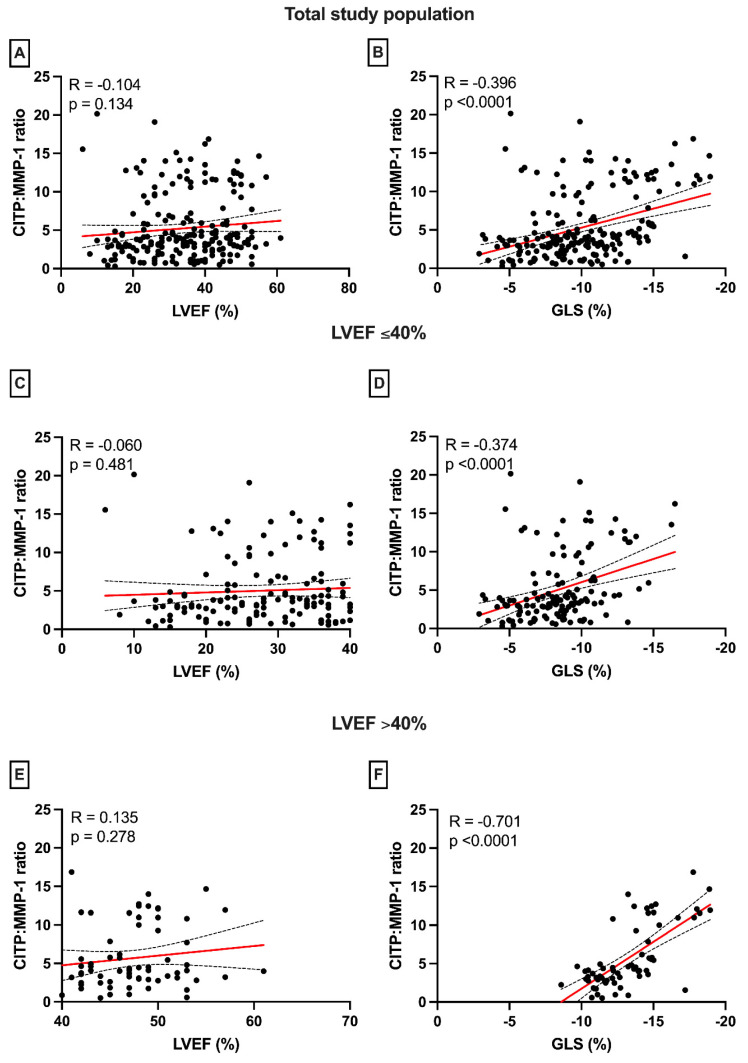
Associations of collagen cross-linking (CITP:MMP-1) with cardiac function parameters (LVEF and GLS). CITP:MMP-1 is not correlated with LVEF in the total study sample, as well as in the subgroups of patients with LVEF above or below 40% (**A**,**C**,**E**). Significant correlations were found between CITP:MMP-1 and GLS in the total study sample (**B**) and in patients with LVEF ≤ 40% (**D**). This correlation is even stronger in patients with mildly reduced LVEF of ≥40% (**F**). Abbreviations: LVEF: left ventricular ejection fraction, GLS: global longitudinal strain, CITP: collagen type I carboxy-terminal telopeptide, MMP-1: matrix metalloproteinase-1. Red line = linear regression line. Dotted lines = 95% CI.

No associations were observed between histological CVF and cardiac function parameters (LVEF: R = −0.000, *p* = 0.998; GLS: R = −0.053, *p* = 0.463). Serum PICP levels and cardiac function parameters (LVEF and GLS) were not significantly correlated in the total study sample (Supplementary Figure S3A,B), in patients with LVEF ≤ 40% (Supplementary Figure S3C,D), or in patients with LVEF ≥ 40% (Supplementary Figure S3E,F). Supplementary Figure S3 is presented as part of the Supplementary Materials, which has been previously published [5]. CITP:MMP-1 was neither correlated with LVEF in the total study sample (Figure 3A) nor in the subgroups with LVEF above and below 40% (Figure 3C,E).

CITP:MMP-1 did significantly correlate with GLS in the total study sample (R = −0.40, *p* < 0.0001, Figure 3B), or in patients with LVEF ≤40% and LVEF > 40% (R = −0.37 and R = −0.70, respectively, both *p* < 0.0001, Figure 3D,E), indicating that higher collagen cross-linking (lower CITP:MMP-1) was associated with worse GLS. No correlations were found between GLS and LGE extent (R = −0.09, *p* = 0.53).

### 3.4. Association of Collagen Biomarkers with Event-Free Survival

During the follow-up of 6 years [5,6,7,8], 47 (22%) patients reached the primary endpoint (all-cause mortality, n = 14; life-threatening arrythmia, n = 19; or HF hospitalization, n = 14). 

The unadjusted Cox regression analysis showed that CITP:MMP-1 (HR 0.985, 95% CI 0.918–1.058, *p* = 0.678) was not associated with a worse outcome, and neither was CITP (HR 1.053, 95% CI 0.892–1.254, *p* = 0.543). However, higher serum MMP-1 levels were associated with a worse outcome (HR 1.026, 95% CI 1.003–1.049, *p* = 0.023). To assess if MMP-1 added further value to our previous risk stratification model [5] including age, sex, LVEF, NYHA class ≥ 3, NTproBNP, PICP, and LGE presence, we added MMP-1 to the model. Here, MMP-1 remained associated with the outcome, after adjustment of the previous covariates (1.026, 95% CI 1.002–1.051, *p* = 0.037, Table 2).

**Figure 4:**
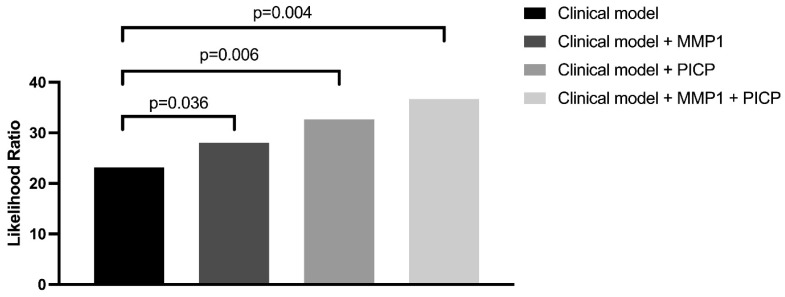
The addition of MMP-1 and PICP improves the goodness-of-fit of the model. MMP-1 and PICP improve the goodness-of-fit as individual markers. Combining MMP-1 and PICP improves the goodness-of-fit even further.

Finally, we evaluated the incremental predictive value of MMP-1 and PICP individually and when combined when it was added to the clinical parameters (age, sex, LVEF, NYHA class ≥ 3, NTproBNP, and LGE presence). The addition of MMP-1 significantly improved the goodness-of-fit (likelihood ratio [LHR] chi-square 28.04, *p* = 0.036). The same applied to the addition of PICP to the same clinical markers (LHR chi-square 32.66, *p* = 0.007). When MMP-1 and PICP were combined and added to the clinical markers, the goodness-of-fit improved even further (LHR chi-square 36.67, *p* = 0.004, Figure 4).

## 4. Discussion

To the best of our knowledge, this is the first study that describes the associations between serum markers of collagen deposition (PICP), collagen cross-linking (CITP:MMP-1), and myocardial contractile function in DCM patients. Our main finding is that a low CITP:MMP-1 ratio, reflecting higher collagen cross-linking, was significantly correlated with impaired longitudinal myocardial contraction (GLS). This correlation was strongest in patients with mildly reduced cardiac function (LVEF > 40%). In addition, higher levels of MMP-1 were independently associated with a worse outcome, whereas the combination of MMP-1 and PICP further improved risk stratification beyond classical risk factors.

### 4.1. The Association of Collagen Biomarkers with Cardiac Function

The imbalance between collagen synthesis and degradation causes the accumulation of type I collagen fibers, resulting in an increased extracellular matrix (ECM) volume and enhanced myocardial stiffness. The alignment of highly cross-linked type I collagen fibers prevents signal and force transmission through the myocardium, resulting in impaired myocardial contraction [19]. Novel techniques enable the detection and refinement of morphological and functional cardiac disorders. CMR-derived feature tracking analysis allows the measurement of myocardial global longitudinal contraction, which is a well-validated marker of cardiac (dys)function [20]. While LVEF is purely based on volumetric changes and predominantly reflects radial contraction, GLS depicts longitudinal shortening [20]. Interestingly, neither CVF nor PICP—both reflecting collagen deposition—were associated with these cardiac function parameters. Similarly, we did not find a correlation between CITP:MMP-1 ratio and LVEF, which is in line with a previous study including DCM patients [21]. A lower CITP:MMP-1 ratio, reflecting higher collagen cross-linking, however, was correlated with impaired longitudinal myocardial contraction (GLS), particularly in DCM patients with mildly reduced LVEF (>40%). This finding is comparable with results from a small study including 38 hypertensive cardiomyopathy patients who also presented with mildly reduced LVEF, showing a correlation between the degree of collagen cross-linking and myocardial contraction [22]. This finding suggests that it is not the extent of collagen deposition but the degree of myocardial cross-linking that is directly associated with longitudinal myocardial contraction. Due to the increased myocardial collagen cross-linking, the end-diastolic muscle fibers might be shortened, resulting in a reduced myocardial longitudinal contraction. Therefore, GLS might mirror the amount of myocardial collagen cross-linking more accurately than LVEF alone [19]. In addition, the increased collagen cross-linking might be the initial cellular compensatory response in an attempt to maintain adequate myocardial contraction and, therefore, LVEF. 

### 4.2. Prognostic Value of Collagen Metabolism-Related Biomarkers

The imbalance between collagen deposition and degradation leading to myocardial fibrosis plays an important role in cardiac dysfunction and impaired clinical outcomes in DCM patients [3]. Higher levels of circulating PICP are associated with adverse outcomes in DCM patients, in addition to the presence of non-ischemic LGE [5]. Besides the quantity of collagen deposition, collagen cross-linking quality also influences prognosis in patients with HF and hypertensive heart disease [4,8,22]. In hypertensive HF patients, the serum CITP:MMP-1 ratio was inversely associated with the risk for HF hospitalization [8] and the combination of an increased cross-linking and a high collagen type I deposition was associated with a higher risk for HF hospitalization and mortality [23]. Until now, the prognostic relevance of collagen cross-linking in DCM patients was unknown, mainly due to the lack of (non-invasive) methods that enable the quantification of cross-linking. While the CITP:MMP-1 ratio was significantly correlated with GLS, it was not directly associated with a worse outcome in the total study sample. This could be explained by the fact that the correlation was strongest in DCM patients with mildly reduced LVEF. It could be hypothesized that the CITP:MMP-1 ratio might be of prognostic importance in this subgroup of patients. Unfortunately, this study was not powered to evaluate this effect in this subgroup, and future studies with larger sample sizes are needed to evaluate this effect. Interestingly, higher levels of circulating MMP-1 were associated with worse prognosis in DCM patients. Moreover, the combination of high MMP-1 and PICP levels predicted a poor outcome in DCM patients, beyond LGE and other well-known predictors of outcome (age, sex, LVEF, NYHA ≥ 3, and NTproBNP). 

### 4.3. Clinical Implications and Future Directions

In recent years, several fibrosis biomarkers have been proposed, among them PICP and CITP:MMP-1 demonstrate robust and reliable associations with histological collagen deposition and cross-linking, respectively [7,24]. Increased levels of circulating fibrosis biomarkers result from either an intrinsic cardiac disease or from systemic, non-cardiac, collagen metabolism. Therefore, these biomarkers should preferably be combined with structural and functional parameters of non-invasive cardiac imaging [4,25]. In this context, the combination of CMR and serum markers of collagen deposition (PICP) and collagen cross-linking (CITP:MMP-1) emerges as a useful tool to improve risk stratification with the potential to track treatment response [26]. Future studies should focus on validating these collagen-metabolism biomarkers as prognostic markers and compiling a risk score containing biomarkers combined with imaging parameters to assess the degree of myocardial fibrotic burden and its effect on myocardial function.

### 4.4. Study Limitations

CMR, blood sampling, and EMB were not performed contemporaneously but over a three-month timespan. This could lead to some biases and could explain the modest (although significant) correlation between the CITP/MMP-1 ratio and GLS in the total study sample. CMR parametric mapping techniques to measure T1 and extracellular volume are recommended as an alternative, non-invasive method to complement circulating fibrosis biomarkers [7]. Unfortunately, T1 mapping has only been adopted in clinical practice in recent years. Therefore, T1 and ECV values are not available in this study population, and future studies are needed to evaluate the direct correlations of collagen biomarkers with CMR-assessed diffuse myocardial fibrosis in DCM. MMPs are proteolytic enzymes containing Zinc ions. Unfortunately, no information regarding Zinc levels or supplementation is available for this study cohort. The relatively low event rate limits the ability to perform extensive multivariable analysis and the power to detect (more subtle) differences in collagen cross-linking biomarkers in this cohort. Nevertheless, we included well-known clinical predictors of prognosis in our adjusted regression models, and PICP and MMP-1 remained independent and incremental predictors of prognosis. Evaluating the optimal prognostic cut-off values of cross-linking biomarkers is beyond the scope of this study. The lack of a validation cohort prevents the validation of our findings. Larger studies with higher event rates that enable more robust parametric modeling are needed for validation and refinement of the presented results, as well as for finding the optimal prognostic cut-off values of these circulating biomarkers.

## 5. Conclusions

Increased collagen cross-linking (CITP:MMP-1 ratio) is associated with impaired myocardial longitudinal contraction (GLS). Collagen deposition and degradation are both of significant importance to determine the effects of myocardial fibrosis on clinical outcomes in DCM patients. The effects of myocardial fibrosis on cardiac function and other clinical outcomes should be assessed using multimodality approaches to fully capture the myocardial fibrotic burden.

## Figures and Tables

**Table 1:** Clinical characteristics of the study sample.

	All (N = 209)
**Demographics**	
Age at diagnosis (years)	54 ± 13 (18–80)
Male (%)	136/209 (65%)
NYHA class III or IV (%)	61/209 (29%)
C-reactive protein	3 (1–7)
NTproBNP	65.9 (22.5–193.5)
**Medical history**	
Hypertension (%)	84/209 (40%)
Diabetes mellitus (%)	21/209 (10%)
Atrial fibrillation (%)	52/209 (25%)
**Medication**	
β-blocker (%)	174/209 (83%)
ACE-inhibitor/ARB (%)	185/209 (89%)
Loop diuretic (%)	112/209 (54%)
Aldosterone antagonist (%)	74/209 (35%)
**Fibrosis biomarkers**	
PICP (ng/mL)	78 (64–102)
CITP (ng/mL)	5.97 (5.33–6.95)
MMP-1 (ng/mL)	5.79 (3.61–9.43)
CITP:MMP-1	3.69 (2.54–6.65)
**Cardiac MRI**	
LVEDVi (mL/m^2^)	136 ± 53
LVESVi (mL/m^2^)	92 ± 50
LVEF (%)	34 ± 12
LV mass index (g/m^2^)	75 ± 27
GLS (n = 203, %)	−10 ± 4
GCS (n = 203, %)	−9 ± 4
GRS (n = 203, %)	17 ± 8
LGE (%)	65/209 (31%)
LGE extent (%)	2.5 [1.1–5.4]
**Endomyocardial biopsy**	
Chronic low-grade inflammation	71/209 (34%)
Collagen volume fraction (%)	7 (4–11)
Time between CMR and EMB (days)	30 (6–50)

Abbreviations: NYHA: New York Heart Association class, NTproBNP: N-terminal pro-B-type natriuretic peptide, ACE: angiotensin-converting enzyme, ARB: angiotensin receptor II blocker, MRI: magnetic resonance imaging, PICP: carboxy-terminal propeptide of procollagen type I, CITP: collagen type 1 fibers, MMP-1: matrix metalloproteinase, LV: left ventricular, EDV: indexed end-diastolic volume, ESVi: indexed end-systolic volume, EF: ejection fraction; GLS: global longitudinal strain, GCS: global circumferential strain, GRS: global radial strain.

**Table 2:** Unadjusted and adjusted Cox regression analyses of collagen cross-linking biomarkers.

	Unadjusted Analysis		Adjusted Analysis *	
	HR (95% CI)	*p*-Value	HR (95% CI)	*p*-Value
CITP	1.053 (0.891–1.245)	0.543	-	-
MMP-1	1.026 (1.003–1.049)	0.023	1.026 (1.002–1.051)	0.037
CITP:MMP-1	0.985 (0.918–1.058)	0.678	-	-

* Adjusted for age, sex, left ventricular ejection fraction, New York Heart Association class ≥ 3, N-terminal pro-B-type natriuretic peptide (log-transformed), carboxy-terminal propeptide of procollagen type I, and presence of late gadolinium enhancement. Abbreviations: HR: hazard ratio per unit increase, CI: confidence intervals, CITP: collagen type I carboxy-terminal telopeptide, MMP-1: matrix metalloproteinase-1.

## Data Availability

The data presented in this study are available on request from the corresponding author. The data are not publicly available.

## Supplementary Materials

The following supporting information can be downloaded at https://www.mdpi.com/article/10.3390/jcm12175695/s1, Figure S1: Flowchart of the study population; Figure S2: Intraobserver variability of GLS; Figure S3: Associations of collagen deposition (PICP) with cardiac function parameters (LVEF and GLS).

## References

[1] Verdonschot J.A.J., Hazebroek M.R., Ware J.S., Prasad S.K., Heymans S.R.B. (2019). Role of Targeted Therapy in Dilated Cardiomyopathy: The Challenging Road Toward a Personalized Approach. J. Am. Heart Assoc..

[2] De Boer R.A., De Keulenaer G., Bauersachs J., Brutsaert D., Cleland J.G., Diez J., Du X., Ford P., Heinzel F.R., Lipson K.E. (2019). Towards better definition, quantification and treatment of fibrosis in heart failure. A scientific roadmap by the Committee of Translational Research of the Heart Failure Association (HFA) of the European Society of Cardiology. Eur. J. Heart Fail..

[3] Díez J., González A., Kovacic J.C. (2020). Myocardial Interstitial Fibrosis in Nonischemic Heart Disease, Part 3/4: JACC Focus Seminar. J. Am. Coll. Cardiol..

[4] Gonzalez A., Schelbert E.B., Diez J., Butler J. (2018). Myocardial Interstitial Fibrosis in Heart Failure: Biological and Translational Perspectives. J. Am. Coll. Cardiol..

[5] Raafs A.G., Verdonschot J.A., Henkens M.T., Adriaans B.P., Wang P., Derks K., Hamid M.A.A., Knackstedt C., Empel V.P., Díez J. (2021). The combination of carboxy-terminal propeptide of procollagen type I blood levels and late gadolinium enhancement at cardiac magnetic resonance provides additional prognostic information in idiopathic dilated cardiomyopathy—A multilevel assessment of myocardial fibrosis in dilated cardiomyopathy. Eur. J. Heart Fail..

[6] Querejeta R., López B., González A., Sánchez E., Larman M., Martínez Ubago J.L., Díez J. (2004). Increased collagen type I synthesis in patients with heart failure of hypertensive origin: Relation to myocardial fibrosis. Circulation.

[7] López B., González A., Ravassa S., Beaumont J., Moreno M.U., San José G., Querejeta R., Díez J. (2015). Circulating Biomarkers of Myocardial Fibrosis: The Need for a Reappraisal. J. Am. Coll. Cardiol..

[8] López B., Ravassa S., González A., Zubillaga E., Bonavila C., Bergés M., Echegaray K., Beaumont J., Moreno M.U., José G.S. (2016). Myocardial Collagen Cross-Linking Is Associated with Heart Failure Hospitalization in Patients With Hypertensive Heart Failure. J. Am. Coll. Cardiol..

[9] Visse R., Nagase H. (2003). Matrix metalloproteinases and tissue inhibitors of metalloproteinases: Structure, function, and biochemistry. Circ. Res..

[10] Maceira A.M., Prasad S.K., Khan M., Pennell D.J. (2006). Normalized Left Ventricular Systolic and Diastolic Function by Steady State Free Precession Cardiovascular Magnetic Resonance. J. Cardiovasc. Magn. Reson..

[11] Ponikowski P., Voors A.A., Anker S.D., Bueno H., Cleland J.G., Coats A.J., Falk V., González-Juanatey J.R., Harjola V.-P., Jankowska E.A. (2016). 2016 ESC Guidelines for the diagnosis and treatment of acute and chronic heart failure: The Task Force for the diagnosis and treatment of acute and chronic heart failure of the European Society of Cardiology (ESC). Developed with the special contribution of the Heart Failure Association (HFA) of the ESC. Eur. J. Heart Fail..

[12] Yancy C.W., Jessup M., Bozkurt B., Butler J., Casey D.E., Drazner M.H., Fonarow G.C., Geraci S.A., Horwich T., Januzzi J.L. (2013). 2013 ACCF/AHA guideline for the management of heart failure: A report of the American College of Cardiology Foundation/American Heart Association Task Force on practice guidelines. Circulation.

[13] Yancy C.W., Jessup M., Bozkurt B., Butler J., Casey D.E., Colvin M.M., Drazner M.H., Filippatos G.S., Fonarow G.C., Givertz M.M. (2017). 2017 ACC/AHA/HFSA Focused Update of the 2013 ACCF/AHA Guideline for the Management of Heart Failure: A Report of the American College of Cardiology/American Heart Association Task Force on Clinical Practice Guidelines and the Heart Failure Society of America. Circulation.

[14] Flett A.S., Hasleton J., Cook C., Hausenloy D., Quarta G., Ariti C., Muthurangu V., Moon J.C. (2011). Evaluation of Techniques for the Quantification of Myocardial Scar of Differing Etiology Using Cardiac Magnetic Resonance. JACC Cardiovasc. Imaging.

[15] Ravassa S., Kuznetsova T., Varo N., Thijs L., Delles C., Dominiczak A., Díez J., Staessen J.A. (2015). Biomarkers of cardiomyocyte injury and stress identify left atrial and left ventricular remodelling and dysfunction: A population-based study. Int. J. Cardiol..

[16] Hazebroek M.R., Moors S., Dennert R., van den Wijngaard A., Krapels I., Hoos M., Verdonschot J., Merken J.J., de Vries B., Wolffs P.F. (2015). Prognostic Relevance of Gene-Environment Interactions in Patients with Dilated Cardiomyopathy: Applying the MOGE(S) Classification. J. Am. Coll. Cardiol..

[17] Caforio A.L.P., Pankuweit S., Arbustini E., Basso C., Gimeno-Blanes J., Felix S.B., Fu M., Heliö T., Heymans S., Jahns R. (2013). Current state of knowledge on aetiology, diagnosis, management, and therapy of myocarditis: A position statement of the European Society of Cardiology Working Group on Myocardial and Pericardial Diseases. Eur. Heart J..

[18] Schneider C.A., Rasband W.S., Eliceiri K.W. (2012). NIH Image to ImageJ: 25 years of image analysis. Nat. Methods.

[19] Pichler G., Redon J., Martínez F., Solaz E., Calaforra O., Andrés M.S., Lopez B., Díez J., Oberbauer R., Adlbrecht C. (2020). Cardiac magnetic resonance-derived fibrosis, strain and molecular biomarkers of fibrosis in hypertensive heart disease. J. Hypertens..

[20] Pedrizzetti G., Claus P., Kilner P.J., Nagel E. (2016). Principles of cardiovascular magnetic resonance feature tracking and echocardiographic speckle tracking for informed clinical use. J. Cardiovasc. Magn. Reson..

[21] Kanoupakis E.M., Manios E.G., Kallergis E.M., Mavrakis H.E., Goudis C.A., Saloustros I.G., Milathianaki M.E., Chlouverakis G.I., Vardas P.E. (2010). Serum Markers of Collagen Turnover Predict Future Shocks in Implantable Cardioverter-Defibrillator Recipients With Dilated Cardiomyopathy on Optimal Treatment. J. Am. Coll. Cardiol..

[22] López B., Querejeta R., González A., Larman M., Díez J. (2012). Collagen cross-linking but not collagen amount associates with elevated filling pressures in hypertensive patients with stage C heart failure: Potential role of lysyl oxidase. Hypertension.

[23] Ravassa S., López B., Querejeta R., Echegaray K., San José G., Moreno M.U., Beaumont F.J., González A., Díez J. (2017). Phenotyping of myocardial fibrosis in hypertensive patients with heart failure. Influence on clinical outcome. J. Hypertens..

[24] López B., Ravassa S., Moreno M.U., José G.S., Beaumont J., González A., Díez J. (2021). Diffuse myocardial fibrosis: Mechanisms, diagnosis and therapeutic approaches. Nat. Rev. Cardiol..

[25] Bayes-Genis A., Aimo A., Jhund P., Richards M., de Boer R.A., Arfsten H., Fabiani I., Lupón J., Anker S.D., González A. (2022). Biomarkers in heart failure clinical trials. A review from the Biomarkers Working Group of the Heart Failure Association of the European Society of Cardiology. Eur. J. Heart Fail..

[26] Ravassa S., López B., Ferreira J.P., Girerd N., Bozec E., Pellicori P., Mariottoni B., Cosmi F., Hazebroek M., Verdonschot J.A. (2022). Biomarker-based assessment of collagen cross-linking identifies patients at risk of heart failure more likely to benefit from spironolactone effects on left atrial remodelling. Insights from the HOMAGE clinical trial. Eur. J. Heart Fail..

