# Supplementary material for: Biomarkers of Collagen Metabolism Are Associated with Left Ventricular Function and Prognosis in Dilated Cardiomyopathy: A Multi-Modal Study
Source: J Clin Med. 2023 Sep 1;12(17):5695. doi: 10.3390/jcm12175695 (PMC10488673; doi:10.3390/jcm12175695)
Supplement: Supplementary file 1 [file jcm-12-05695-s001.zip › jcm-2528428-supplementary.pdf]

Supplemental Figure S1. Flowchart of the study population

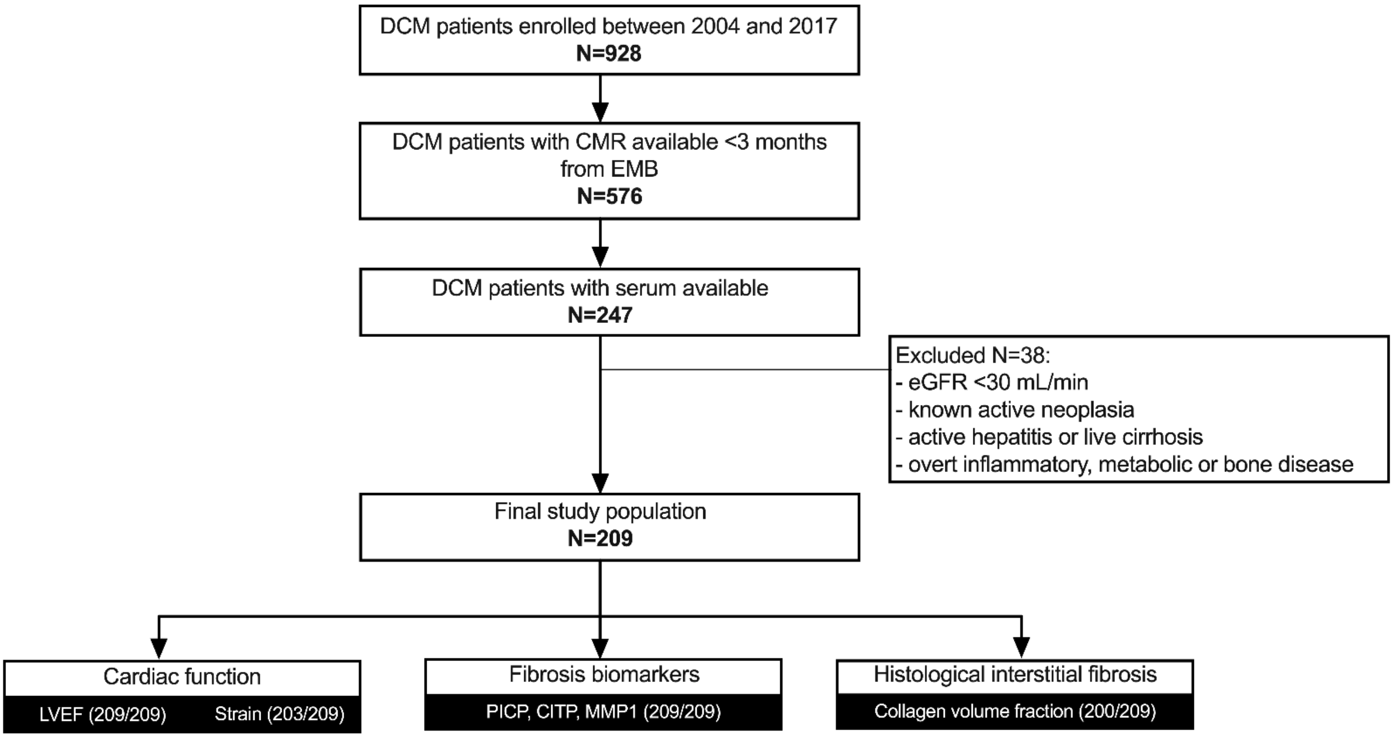

Abbreviations: DCM: dilated cardiomyopathy, CMR: cardiac magnetic resonance imaging, GFR: glomerular filtration rate, LVEF: left ventricular ejection fraction, PICP: carboxy-terminal propeptide of procollagen type I, CITP: collagen type 1 fibers, MMP1: matrix metallo-proteinase.

Supplemental Figure S2. Intraobserver variability of GLS

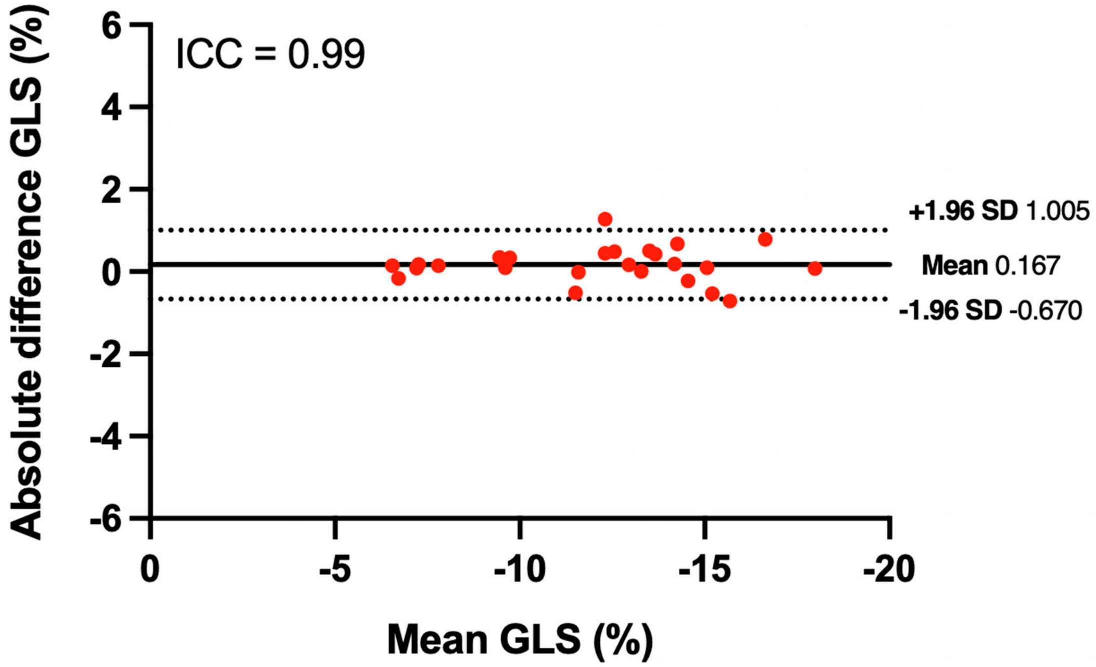

Abbreviations: GLS = global longitudinal strain, ICC = interclasss correlation coefficient, SD = standard deviation.

Supplemental Figure S3. Associations of collagen deposition (PICP) with cardiac function parameters (LVEF and GLS)

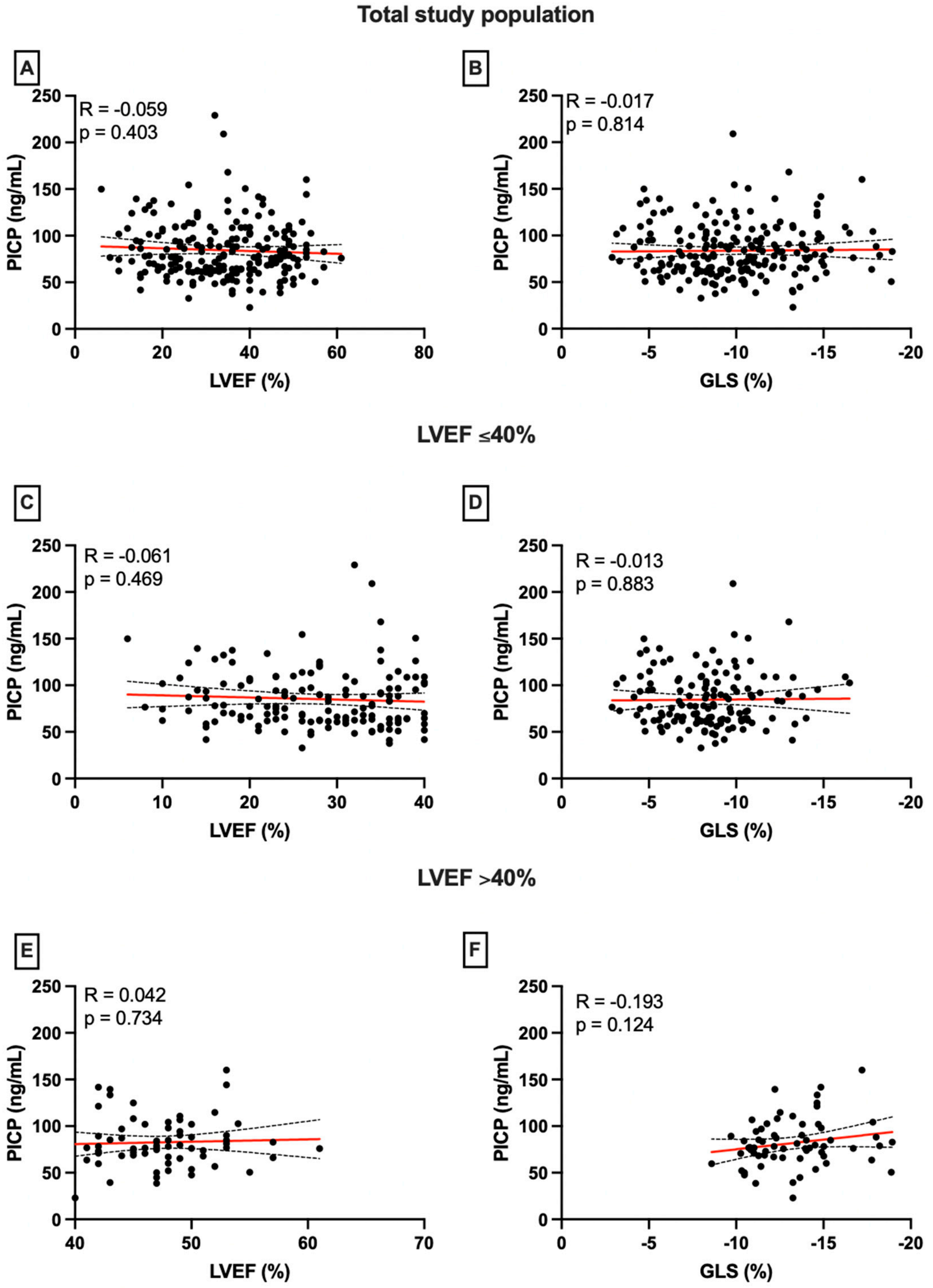

PICP was not correlated with either LVEF or GLS.
Abbreviations: LVEF: left ventricular ejection fraction, GLS: global longitudinal strain, PICP: carboxy-terminal propeptide of procollagen type I.
